# Population Changes in a Community of Alkaliphilic Iron-Reducing Bacteria Due to Changes in the Electron Acceptor: Implications for Bioremediation at Alkaline Cr(VI)-Contaminated Sites

**DOI:** 10.1007/s11270-015-2437-z

**Published:** 2015-05-13

**Authors:** Samuel J. Fuller, Ian T. Burke, Duncan G. G. McMillan, Weixuan Ding, Douglas I. Stewart

**Affiliations:** School of Civil Engineering, University of Leeds, Leeds, LS2 9JT UK; School of Earth and Environment, University of Leeds, Leeds, LS2 9JT UK; University Hospital Jena, Friedrich-Schiller University, 07743 Jena, Germany; School of Process, Environmental and Materials Engineering, University of Leeds, Leeds, LS2 9JT UK

**Keywords:** COPR, Bacteria, Alkaliphile, Chromium, Iron, Bioremediation

## Abstract

A serial enrichment culture has been grown in an alkaline Fe(III)-citrate-containing medium from an initial inoculum from a soil layer beneath a chromium ore processing residue (COPR) disposal site where Cr(III) is accumulating from Cr(VI) containing leachate. This culture is dominated by two bacterial genera in the order Clostridiales, *Tissierella*, and an unnamed *Clostridium XI* subgroup. This paper investigates the growth characteristics of the culture when Cr(VI) is added to the growth medium and when aquifer sand is substituted for Fe(III)-citrate. The aim is to determine how the availability and chemical form of Fe(III) affects the growth of the bacterial consortium, to determine the impact of Cr(VI) on growth, and thus attempt to understand the factors that are controlling Cr(III) accumulation beneath the COPR site. The culture can grow fermentatively at pH 9.2, but growth is stronger when it is associated with Fe(III) reduction. It can withstand Cr(VI) in the medium, but growth only occurs once Cr(VI) is removed from solution. Cr(VI) reduced the abundance of *Tissierella sp*. in the culture, whereas the *Clostridium XI* sp. was Cr(VI) tolerant. In contrast, growth with solid phase Fe(III)-oxyhydroxides (present as coatings on aquifer sand) favoured the *Tissierella C* sp., possibly because it produces riboflavin as an extracellular electron shuttling compound allowing more efficient electron transfer to solid Fe(III) phases. Thus, it is suggested that bacterially mediated Cr(III) reduction in the soil beneath the COPR site is dependent on Fe(III) reduction to sustain the bacterial community.

**Electronic supplementary material:**

The online version of this article (doi:10.1007/s11270-015-2437-z) contains supplementary material, which is available to authorized users.

## Introduction

Many industrial processes produce highly alkaline wastes that are contaminated with toxic trace metals/metalloids (e.g. coal combustion, lime production, chromium ore processing, cement production, iron and steel manufacture, and bauxite refining to name but a few: Burke et al. 2013; Deakin et al. 2001; Jankowski et al. 2006; Mayes and Younger 2006). Until recently, these bulk wastes were often poorly disposed of, frequently in unlined tips (Higgins et al. 1998; Mayes et al. 2008; Stewart et al. 2006). When rainwater infiltrates the waste, it can leach the toxic metals, transporting them into the groundwater beneath disposal sites. The subsequent fate of these toxic metals then depends on the biogeochemical reactions that take place when the alkaline leachate passes through the soil beneath the site.

Microbial Fe(III)/Fe(II) redox cycling can have a major impact on the solubility and mobility of many trace metal/metalloid contaminants in the geosphere, particularly those that form stable oxyanions such as Cr, V, Mo, W, Tc and Se (Lovley 2001). Neo-formed Fe(II) phases can reduce the oxidation state of some these contaminants (e.g. vanadate, pertechnetate, chromate, selenite; Burke et al. 2006; Charlet et al. 2007; White and Peterson 1996; Whittleston et al. 2011), while others form surface complexes on hydrated Fe(II) minerals (e.g. molybdate, tungstate; O’Loughlin et al. 2010; Rey and Reggiani 2005). As a result, the contaminant is less mobile and, in some cases, less toxic (Veeramani et al. 2011). Bioremediation, by exploiting indigenous iron-reducing soil bacteria, has therefore been suggested as an elegant and relatively inexpensive method to reduce the mobility of these contaminants in the subsurface (Anderson et al. 2003; NABIR 2003).

During dissimilatory Fe(III) reduction, bacteria harvest electrons from organic carbon (the electron donor) and transfer them to the Fe(III) substrate (the electron acceptor) which is reduced (Liermann et al. 2007). This process generates an electrochemical gradient across the cell membrane (the “proton motive force”) which can be used to drive internal processes such as substrate import and ATP synthesis (Kim and Gadd 2008; Madigan et al. 2003; McMillan et al. 2009; Mitchell 1961). Bacteria use several different mechanisms to reduce Fe(III). These include intracellular reduction of soluble Fe(III) (Glasauer et al. 2007), reduction by surface contact with extracellular Fe(III) (Bae and Lee 2013; Lovley 2008; Shi et al. 2006; White et al. 2013), and extracellular reduction of Fe(III). Extracellular reduction requires a mechanism for transferring electrons between the cells to the iron source. This can involve cells excreting of electron shuttling compounds, such as quinones and flavins (Bae and Lee 2013; Okamoto et al. 2014; von Canstein et al. 2008), opportunistic use of soluble humic substances (Lovley et al. 1998), or the use of conductive appendages that extend from the cell walls (so called ‘nanowires’; Gorby et al. 2006).

Some trace metals, including Cr(VI), Tc(VII), V(V), Mo(VI) and U(VI), can be reduced directly as part of microbial metabolism (Abdelouas et al. 1998; Burke et al. 2006; Carpentier et al. 2005; Harish et al. 2012; Lloyd et al. 2000; Lovley 1993; Shukor et al. 2010). However, there is much debate about whether these compounds can support life as the sole electron acceptors for dissimilatory metabolism. For example, it is relatively common for bacteria to enzymatically reduce Cr(VI) in both aerobic and anaerobic conditions (*Enterobacter cloacae*; Harish et al. 2012; *Shewanella putrefaciens* and *Shewanella alga*; Liu et al. 2002; *Pyrobaculum islandicum*; Lloyd and Lovley 2001; *Desulfovibrio vulgaris*; Lovley and Phillips 1994; e.g. *Escherichia coli*; Shen and Wang 1993). However, very few bacteria strains are able to use Cr(VI) as the sole electron acceptor on which to respire, as it can pass through the cell membrane via the sulphate transport system, and it is toxic to many bacteria (Daulton et al. 2007). Once inside the cell wall, it can readily react with DNA and other intercellular agents that are subsequently damaged (Cervantes and Silver 1992; Dhal et al. 2013; Wang and Shen 1997). This suggests the possibility that, although some bacteria can reduce Cr(VI), the act of reduction can damage the cell leading to mutations and cell death.

Some bacterial species have been isolated from alkaline soils and sediments that can respire using acetate as an electron donor and Fe(III) as an electron acceptor when the pH > 9 (Roh et al. 2007; Zavarzina et al. 2006). However, high pH is a challenging environment for dissimilatory metal-reducing bacteria as it is difficult to maintain a proton motive force across the cell membrane when the external pH exceeds that of the cytoplasm. This may favour fermentative metabolism over respiration in highly alkaline conditions. Fermentative bacteria do not need to generate a proton motive force to drive cellular processes. Instead, they can harvest energy from an internally balanced process in which a fermentable organic substrate is both oxidised and reduced (Madigan et al. 2003; Schmitz et al. 2006). ATP is synthesised in the cytoplasm by substrate-level phosphorylation (Barker 1981; Nelson and Cox 2013). Redox balance must be maintained (which places constraints on the amount of energy that can be obtained), and H_2_ production is one means of disposing of excess electrons (Gottschalk 1986; Kim and Gadd 2008). Some fermentative alkaliphiles in the order Clostridiales have been demonstrated to indirectly reduce extracellular iron by dumping electrons to Fe(III) as an alternative method of maintaining internal redox balance within cells (Dobbin et al. 1999; Garnova et al. 2003; Gorlenko et al. 2004; Kevbrin et al. 1998).

Recently, soil samples were recovered from directly below a nineteenth century COPR disposal site in West Yorkshire. Due to a perched water table in the COPR, water that has a pH value >12 and contains approximately 1 mmol L^−1^ Cr(VI) was seeping downwards into these soils (Whittleston et al. 2011; Fuller et al. 2014). However, geochemical analysis showed that there was rapid attenuation of Cr(VI) with distance from the waste tip and accumulation of Cr(III) within the organic rich former topsoil layer immediately beneath the tip (Whittleston 2011). Within this soil layer, a large proportion of the 0.5 N HCl extractable Fe (a proxy for microbially available Fe; Weber et al. 2001) was in the Fe(II) oxidation state. The persistence of the Fe(II) oxidation state in the presence of a Cr(VI) flux suggests that microbial iron reduction was occurring (Stewart et al. 2010). X-ray absorption spectroscopy analysis of soil samples indicated that Cr is present as a mixed Cr(III)–Fe(III) oxyhydroxide phase, suggesting that the elevated soil Cr content is due to reductive precipitation of Cr(VI) by Fe(II) (Whittleston et al. 2011). Bacteria cultivated from this soil horizon have been repeatedly grown in alkaline anaerobic medium containing yeast extract and Fe(III) citrate. Growth of the community resulted in the release of riboflavin to the medium, most probably to mediate extracellular electron transfer, and the accumulation of Fe(II)-containing phases in spent medium (Fuller et al. 2014).

Previous work has therefore shown that the fate of toxic Cr(VI) released from alkaline COPR depends on the biogeochemical reactions that take place when the leachate interacts with soils at the site and particularly on microbial Fe(III)/Fe(II) redox cycling. This paper takes the community of bacteria recovered by Whittleston from soil beneath COPR disposal site and investigates Cr(VI) reduction in an alkaline anaerobic medium containing Cr(VI) and Fe(III) as electron acceptors, with aquifer sand as a source of solid phase Fe(III) and with Cr(VI) as the major electron acceptor. The specific objectives are to determine how the availability and chemical form of Fe(III) affect the growth and community structure of the bacterial consortium and determine the impact of an increasing concentration of Cr(VI) on these properties. Understanding how Fe(III) and Cr(VI) reduction processes can be coupled in COPR-contaminated soils will be discussed with regard to potential bioremediation strategies at such sites.

## Materials and Methods

### Alkaliphilic Fe(III)-Reducing Bacterial Community

The bacterial community that was used as an inoculum for experiments reported in this study was originally recovered from the former topsoil horizon immediately beneath a nineteenth century COPR tip (see Whittleston 2011; Whittleston et al. 2011; for details). Since its recovery, the bacteria community used in this study has been repeatedly grown anaerobically in alkaline growth medium containing aqueous Fe(III) (“Fe(III)-citrate medium”: details given below). Previous studies have shown that the population of this continuously transferred enrichment culture is stable and contains bacteria of the genera *Tissierella*, *Alkaliphilus* and the *Clostridium XI* subgroup containing *Colletotrichum mangenotii*, which could be further classified into five operational taxonomic units (OTUs), from now on referred to as *Tissierella* A (7 % of the population), *Tissierella* B (5 %), *Tissierella* C (36 %), *Alkaliphilus* (8 %) and *Clostridium XI* (44 %) (GenBank numbers KF362050-KF362117; Fuller et al. 2014).

### Alkaline Growth Media

The basal medium used throughout this study was a semi-defined growth medium that contained NaH_2_PO_4_·H_2_O (0.356 g L^−1^), KCl (0.1 g L^−1^), 10 mL L^−1^ each of standard vitamin and mineral mixtures (Bruce et al. 1999), and yeast extract (2 g L^−1^) as a source of energy and carbon. Fe(III)-citrate medium was made by adding Fe(III) citrate (2 g L^−1^) to the basal medium as a terminal electron acceptor. The pH value of the medium was buffered to 9.2 by the addition of Na_2_CO_3_ (1 M carbonate solution). The medium was boiled for 30 min and then purged with nitrogen for 30 min to exclude oxygen. One hundred millilitre aliquots of medium were placed in 100-mL glass serum bottles, and the remaining headspaces were filled with N_2_. The bottles were sealed with butyl rubber stoppers with aluminium crimps, and heat sterilised at 120 °C for 20 min. Fe in the Fe(III)-citrate medium remained soluble as a red-coloured Fe(III)-citrate complex, but the medium also contained a small amount of a hydrous ferric oxyhydroxide precipitate which formed when the pH was adjusted to pH 9.2.

Cr(VI) medium was prepared using the same protocol as Fe(III)-citrate medium except that the Fe(III)-citrate was omitted and K_2_CrO_4_ (39 mg L^−1^; 200 μmol L^−1^) was added to the basal medium as an electron acceptor. Similarly, FeCr medium was made by adding both Fe(III) citrate (2 g L^−1^) and Cr(VI) to the basal medium. The amount of K_2_CrO_4_ used varied between 100 and 8500 μmol L^−1^ (from 19.5 to 1657.5 mg L^−1^). Henceforth, the amount of K_2_CrO_4_ (μmol L^−1^) will be indicated by the number included in the medium name (e.g. FeCr200 contained 200 μmol L^−1^ of Cr(VI).

Aquifer sand medium was prepared by adding alluvial sand to the basal medium (final concentration 100 g L^−1^). This sand was recovered from beneath the nineteenth century COPR waste tip (Stewart et al. 2010). It is orange in colour, uniformly granular and was sieved, so only particles less than 0.5 mm were used. X-ray diffraction (XRD) analysis of the sand indicated it to be predominantly quartz with a little kaolinite. It contained 5 % Fe and 0.5 % Mn as determined by X-ray fluorescence (XRF). This solid-phase Fe(III) is present as fine particles and grain coatings.

Growth of the bacterial community in the absence of Fe(III) was investigated using the basal medium, and the basal medium supplemented with tri-sodium citrate (2 g L^−1^).

### Growth Characterisation

Growth medium bottles were inoculated with 1 % medium containing bacteria in the upper exponential phase of growth (the inoculum typically contained ∼200 cells nL^−1^). Bottles were kept at a constant temperature of 21 ± 1 °C. The bottles were shaken by hand and sampled (2 mL) using sterile 19 gauge needles and aseptic, anaerobic technique (Burke et al. 2006). Cell numbers in growth media samples were determined by direct counting using an improved Neubauer haemocytometer viewed under an Olympus BH-2 optical microscope. HCl-extractable Fe(II) was determined by adding 0.5 mL of sample to 2 mL of 0.5 N HCl. One hundred micorlitres was then placed in a disposable 1.5-mL cuvette and mixed with 900 μL diH_2_O and 100-μL ferrozine solution (Lovley and Phillips 1986). This was then mixed by inversion, the colour allowed to develop for 10 min, and the absorbance at 562 nm read using a Thermo Scientific BioMate 3 UV/VIS Spectrophotometer. Aqueous Cr(VI) concentration was measured by first mixing 100-μL centrifuged sample, 900 μL of 10 mmol L^−1^ H_2_SO_4_ and 100 μL diphenylcarbazide solution in a disposable 1.5-mL cuvette by inversion (USEPA 1992). The colour was left to develop for 10 min and the absorbance measured at 540 nm. A Hach HQ40d pH meter was used to measure pH.

### 16S rRNA Gene Sequencing

The procedures used to extract bacterial DNA and to sequence the 16S rRNA gene have been reported previously (Fuller 2013). Briefly, DNA was recovered from the growth medium bottles using a FastDNA spin kit for soils (MP Biomedicals, USA). The bottles were shaken, and 15 mL of suspension was extracted using aseptic technique. Samples were centrifuged at 1800×*g* for 15 min, the supernatant removed and the pellet suspended in 978-μL sodium phosphate buffer as supplied with the FastDNA kit (the kit manufacturer’s protocol was then followed). The eluted DNA was analysed on a 1 % *w*/*v* agarose/TBE gel containing ethidium bromide, and the area containing DNA fragments between 3000 and 20,000 bp in length was recovered. The DNA was extracted from the gel using a QIAquick gel extraction kit (QIAGEN ltd, Germany).

Polymerase chain reaction (PCR) was used to amplify a 1.5-kb fragment of the 16 s rRNA gene using the broad specificity primers 8f (AGAGTTTGATCCTGGCTCAG) (Eden et al. 1991) and 1492r (ACGGYTACCTTGTTACGA, where *Y* = *C* or *T*) (Weisburg et al. 1991) or 1525r (AAGGAGGTGWTCCARCC) (Lane 1991). The PCR products were viewed using an agarose/TBE gel, and the desired product was excised and extracted from the gel. This was then ligated into the pGEM-T easy (Promega Corp., USA) cloning vector and transformed into XL1-blue competent *E. coli* cells (Agilent Technologies UK Ltd). The cells were grown on LB-agar plates containing 100 μg mL^−1^ ampicillin. Blue/white colour screening was employed to select viable colonies which were subsequently sent to GATC Biotech Ltd (Germany) for sequencing.

Sequences were checked for chimeras using Mallard (v1.02) (Ashelford et al. 2006), and non-chimeric sequences were grouped into operational taxonomic units (OTUs) using MOTHUR (v1.30.2) (>98 % nearest neighbour sequence similarity cut-off) (Schloss et al. 2009). Sequences from each sample were grouped into OTUs separately before a study-wide analysis was conducted to compare OTUs between samples. Sequences were classified using the Ribosomal Database Project (RDP) naïve Bayesian Classifier using an 80 % bootstrap cut-off value (Wang et al. 2007). This bootstrap cut-off value results in 98.7 % of sequences being correctly classified to genus level (Claesson et al. 2009). All sequences were submitted to the GenBank database with accession numbers KF797922–KF798171.

### Isolation and Quantification of Soluble Electron-Shuttling Compounds

This was conducted in a similar method to that described in Fuller et al. (2014) and McMillan et al. (2010). One hundred millilitres of early stationary phase culture suspension was centrifuged at 9000×*g* for 15 min to separate cells from the growth medium. Culture supernatant was neutralised with HPLC-grade HCl to pH 7 and extracted with 100 mL of ethyl acetate. The bottom aqueous layer was discarded. The pooled organic phase was transferred into an acid-cleaned high-density polyethylene (HDPE) bottle, and residual water was removed by drying over sodium sulphate (5 g) at 4 °C overnight. The organic phase was then filtered through 0.45-μm polytetrafluoroethylene (PTFE) syringe filter (Sartorius) and desiccated using a rotary evaporator. The resulting residue was dissolved with MilliQ H_2_O in an ultrasonic bath (Elma, Elmasonic S30).

A 10-mL column containing 8-g XAD-16 resin (Sigma) was pre-cleaned with 100 % methanol and rinsed thoroughly with deionised H_2_O. The ethyl acetate soluble fraction extract was slowly transferred onto the column (XAD-16 is a non-ionic macroreticular resin that is designed to adsorb organic substances up to 40,000 MW from aqueous systems and polar solvents by hydrophobic and polar interactions). Compounds that bound to the resin were eluted sequentially with four bed volumes of 10, 50 and 100 % methanol (HPLC grade Merck). The 50 and 100 % elutions were pooled and reduced to ∼10 mL using a rotary evaporator at <30 °C. This solution was then transferred to a 15-mL test tube and desiccated by speedvac (Savant SC210A). The resulting dark orange residue was suspended in either 20 mmol L^−1^ 3-(*N*-morpholino) propanesulfonic acid (MOPS), 30 mmol L^−1^ Na_2_SO_4_ pH 7.4 or deionised H_2_O for further spectroscopy and quantification. Unused Fe(III)-citrate medium was subjected to the same extraction and used as a control. Flavin quantification was performed by scanning wavelengths from 300 to 700 nm using a UV-2 UV/Vis spectrophotometer (Unicam). A standard curve was generated by observing known concentrations (0.05, 0.125, 0.25, 0.5, 1 μmol L^−1^) of riboflavin. An extinction coefficient at 455 nm (*ε* = 12,500 cm^−1^ M^−1^) was used to quantify concentration (Otto et al. 1981).

Fluorescence spectra of purified culture supernatant were measured on a Quanta Master 30 (PTI/Photomed) fluorescence spectrometer using a 1-cm path length. Slit widths of 0.5 and 1.5 mm were used for excitation and emission wavelengths, respectively.

## Results

### Bacterial Growth in Fe(III)-Citrate Medium

**Fig. 1:**
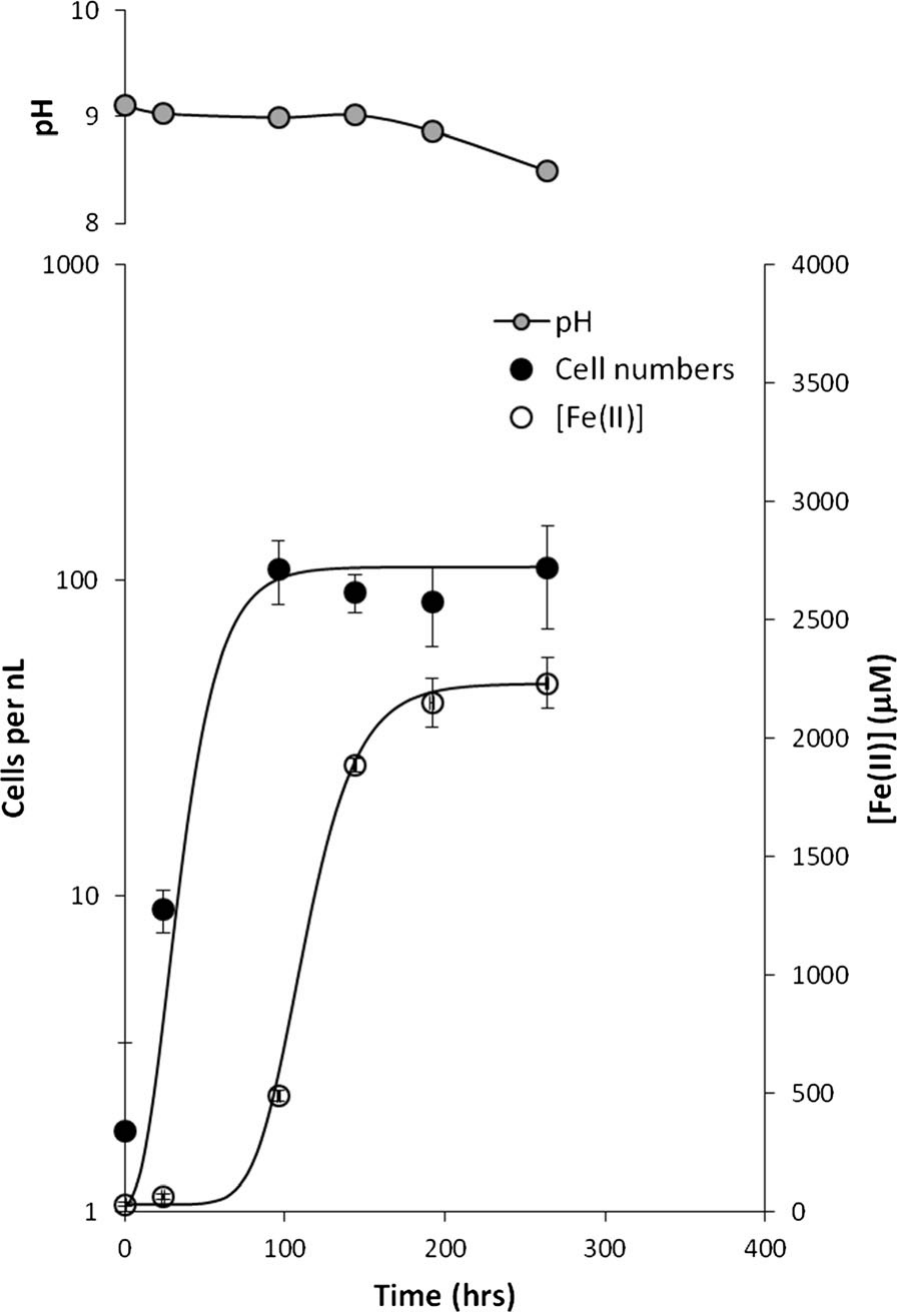
Growth of alkaliphilic Fe(III)-reducing community in Fe(III)-citrate medium. *Error bars* indicate one standard deviation from the mean of triplicate results. Sigmoidal growth curves have been fitted to the cell count and Fe(II) data (Zwietering et al. 1990)

Growth of the alkaliphilic iron-reducing enrichment culture in Fe(III)-citrate medium started shortly after transfer of the inoculum into fresh medium, with very little time lag (Fig. 1). Cell numbers increased exponentially to a maximum of just over 100 cells nL^−1^ at ∼100 h, when the cell numbers reached the stationary phase (standard sigmoidal growth curves are fitted to the data: Zwietering et al. 1990). The total Fe(II) concentration in the growth bottles showed a very similar sigmoidal trend, but the reduction of Fe(III) to Fe(II) lagged behind the increase in cell numbers and was most rapid as cell numbers peaked. Iron reduction was accompanied by a reduction in pH from 9.1 to ∼8.5. These growth characteristics are substantively the same as those reported by Fuller et al. (2014), although they observed a slightly longer lag phase before the initial growth in cell numbers.

**Fig. 2:**
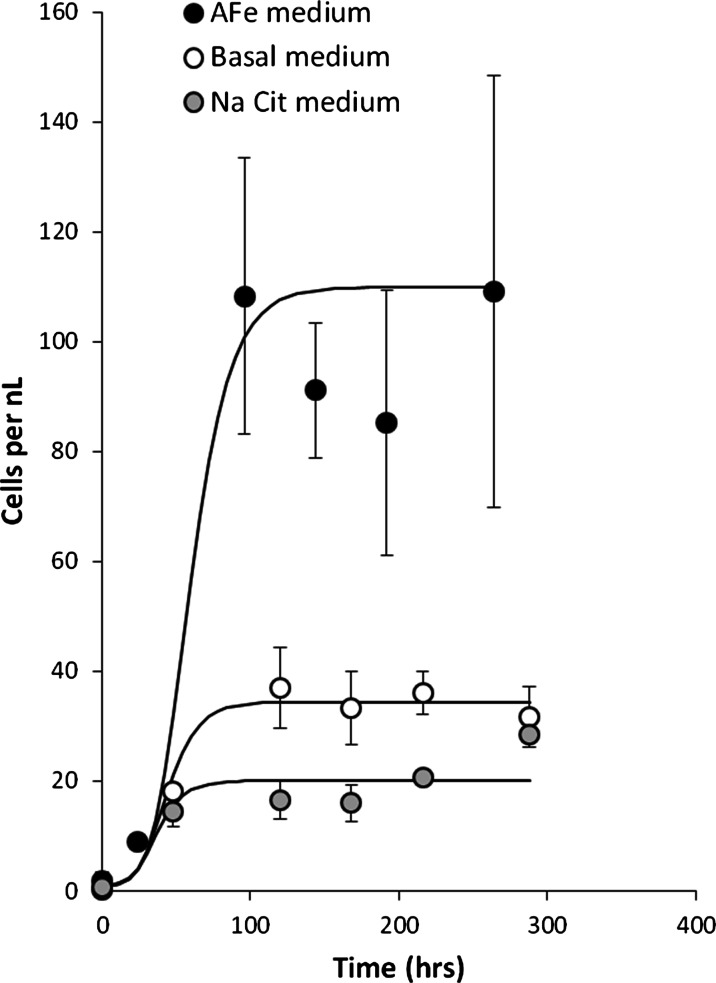
Growth of alkaliphilic Fe(III)-reducing community in basal, sodium citrate and Fe(III)-citrate media. *Error bars* indicate one standard deviation from the mean of triplicate results

When the bacterial community was grown in the basal medium and the basal medium supplemented with sodium citrate (2 g L^−1^), there were similar short lag phases followed by growth in cell numbers (Fig. 2). However, when the stationary phase was reached, cell numbers in these media without Fe(III) were ∼one third the number in the Fe(III)-citrate medium at the same growth stage (a linear scale has been used in Fig. 2 for cell numbers for ease of comparison). Final cell numbers were slightly higher in the basal medium than in the medium supplemented with sodium citrate, indicating that it is the Fe(III) and not the citrate that has a beneficial effect on the growth characteristics of the bacterial community.

### Bacterial Growth and Metal Reduction in Media Containing Both Fe(III) and Cr(VI)

**Fig. 3:**
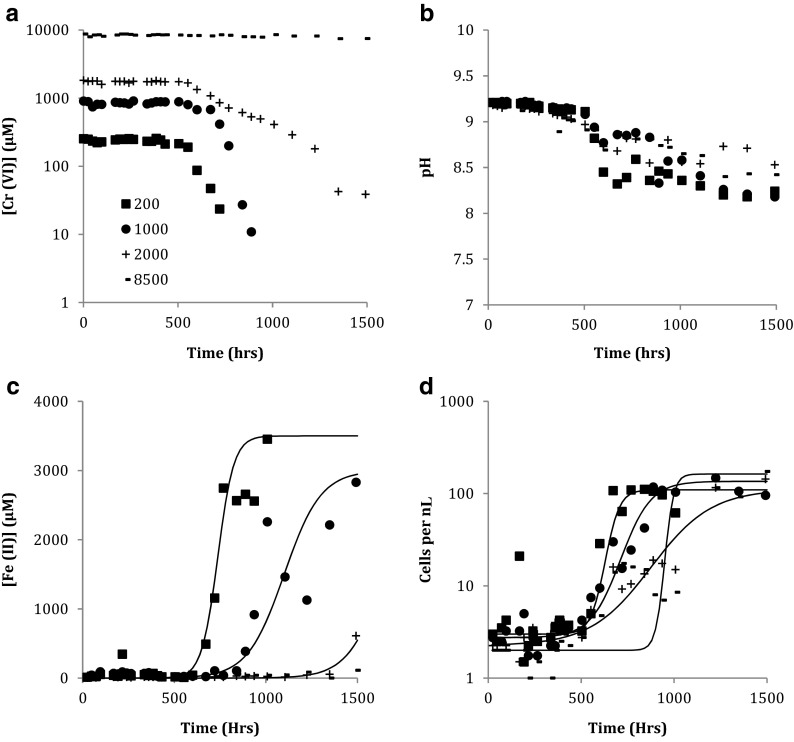
Growth of alkaliphilic Fe(III)-reducing community in FeCr medium with initial Cr(VI) concentrations of 200, 1000, 2000 and 8500 μmol L^−1^. **a** Cr(VI) concentration with time. **b** Total Fe(II) concentration with time. **c** Cell numbers with time. **d** pH with time. Sigmoidal growth curves have been fitted to the variation cell numbers and Fe(II) concentration with time (Zwietering et al. 1990)

Bacteria in the late exponential phase of growth were taken from Fe(III)-citrate medium and inoculated into FeCr medium. When the initial Cr(VI) concentration in the FeCr medium was between 100 and 2000 μmol L^−1^, Cr(VI) was removed from the aqueous phase of the medium. This started after ∼500 h, but the time taken for complete Cr(VI) removal increased with the initial Cr(VI) concentration, taking from 100 to 1000 h (Fig. 3a, Supplementary Information Fig. S1A). The removal of Cr(VI) from solution was followed by an increased Fe(II) concentration (Fig. 3c, S1C). Thus, the time taken before Fe(II) production commenced increased with increasing initial Cr(VI) concentration. In all these tests, an increase in cell numbers was closely linked to the start of Fe(II) production.

When the initial Cr(VI) concentration in the FeCr medium was 4500 and 8500 μmol L^−1^ Cr(VI) removal from solution started after 720 and 1224 h, respectively. After 1500 h, the Cr(VI) concentrations were 2800 and 7500 μmol L^−1^. In these experiments, an increase in cell numbers commenced after ∼1000 h, before all the Cr(VI) was removed from solution and before any Fe(II) was produced (Fig. 3d, S1D).

In all the tests, the pH value of the FeCr medium remained fairly constant at 9.2 for ∼400 h before decreasing slightly to an average final pH value of 8.5 after 1500 h (Fig. 3b, S1B).

### Bacterial Growth in Cr(VI) Medium

Bacteria in the late exponential phase of growth were taken from Fe(III)-citrate medium and grown repeatedly in Cr(VI) medium (this contained 200 μmol L^−1^ Cr(VI)). Colour change of the medium from yellow to clear indicated that Cr(VI) removal from the aqueous was occurring (spectroscopic measurements on the cleared medium from selected bottles confirmed that Cr(VI) < 20 μmol L^−1^). The bacterial community was able to sustain weak growth accompanied by Cr(VI) reduction for 10 repeat subculture cycles before Cr(VI) removal ceased (see Table 1 for success rates). In growth positive tests, the time taken for complete Cr(VI) removal from the aqueous phase was about 25 days. Once growth of subsequent subcultures failed, an aliquot of medium from the seventh growth cycle was inoculated back into Fe(III)-citrate medium and grown-on repeatedly. Sustained growth in Fe(III)-citrate medium was observed; however, 16S rRNA gene sequencing (reported below) showed that repeated growth in Cr(VI) medium had applied a selective pressure to the community. Therefore, the revived bacterial community will be referred to as the Cr(VI) medium community.

**Table 1:** Record of growth of bacterial community in Cr(VI) medium

Growth Cycle	1	2	3	4	5	6	7	8	9	10
Outcome	+++	++−	+−−	++−	+−−	+−−	+−−	++−	++−	−−−

+ denotes final aqueous Cr (VI) concentration <10 % of the initial concentration, − indicates no change in the aqueous Cr (VI) concentration

### Bacterial Growth in Aquifer Sand Medium

**Fig. 4:**
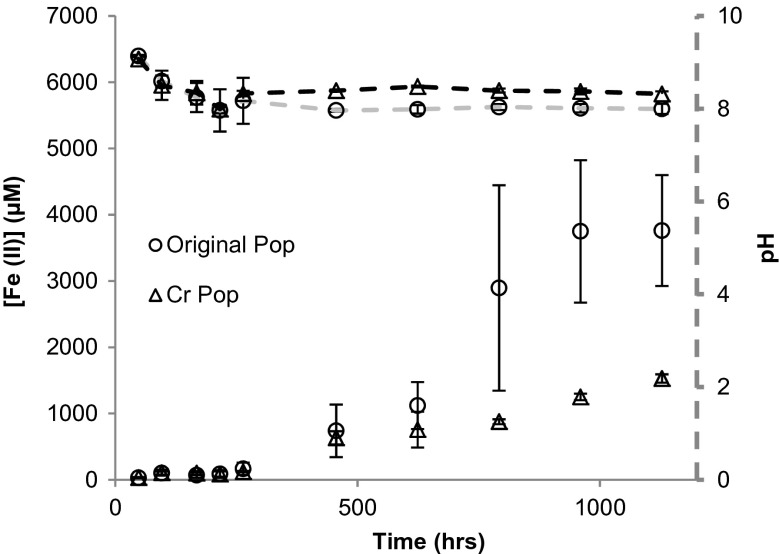
Average Fe(II) concentration and pH for bacteria grown in aquifer sand medium. *Circle* denotes bacterial inoculum from the original Fe(III)-citrate medium. *Triangle* denotes bacterial inoculum from Cr(VI) medium. *Error bars* indicate one standard deviation from the mean

The alkaliphilic Fe(III)-reducing community and the Cr(VI) medium community were inoculated into aquifer sand medium (this medium contained Fe in the coatings on sand grains and as fine particles). Initially, the amount of Fe(II) present was very low because the Fe in the aquifer sand was predominantly in the Fe(III) oxidation state (Fig. 4). In both systems, there was an increase in the amount of Fe(II) after 250 h. In the system inoculated with the alkaliphilic Fe(III)-reducing community, the amount of Fe(II) increased to ∼4000 μmol L^−1^ after ∼1000 h. In the system inoculated with the Cr(VI) medium community, the amount of Fe(II) increased to ∼1500 μmol L^−1^ after ∼1200 h. In both systems, the average pH dropped from 9.2 to ∼8 during the first 250 h and then remained steady, while the amount of Fe(II) increased.

### Sequencing of the Bacteria Population

**Fig. 5:**
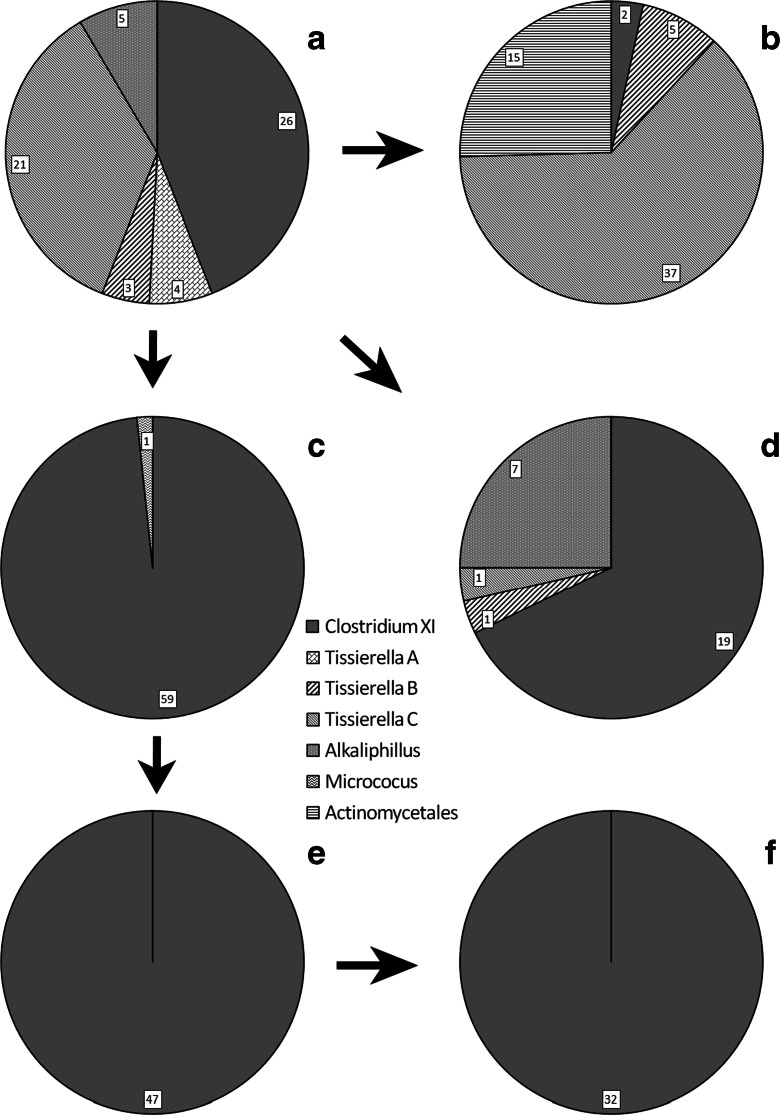
*Pie charts* showing the percentage of the bacterial population assigned to each OTU from **a** Fe(III)-citrate medium. **b** Bacteria from **a** grown in aquifer sand medium. **c** Bacteria from A grown repeatedly in Cr(VI) medium. **d** Bacteria from A grown in FeCr200 medium. **e** Bacteria from C grown in Fe(III)-citrate medium. **f** Bacteria from E grown in aquifer sand medium

Cloning and sequencing of the bacteria from the FeCr200 medium produced 28 sequences from the three different genera of bacteria presented. MOTHUR analysis indicated that there were four OTUs (based on >98 % nearest neighbour similarity) which were the same as OTUs found in the original bacterial community (see Fig. 5). There were single Tissierella B and Tissierella C sequences, 19 Clostridium XI sequences and seven *Alkaliphilus* sequences (the representative sequences of each OTU from this population had the GenBank numbers KF797990, KF797997, KF798001 and KF797986, respectively).

Sixty sequences were obtained from the Cr(VI) medium, and 59 were from genus Clostridium XI (representative sequence KF797963). These belonged to the same OTU as the Clostridium XI in the original bacterial community. One sequence was from genus Micrococcus (sequence KF797945, which has a 97 % RDP similar score when compared to *Micrococcus luteus*). When this population was inoculated back into Fe(III)-citrate medium, all 47 sequences were Clostridium XI (representative sequence KF798082). When it was then grown on solid phase Fe(III), all 32 sequences were Clostridium XI (representative sequence KF798143). In both cases, these sequences were part of the same OTU as the Clostridium XI in the original bacterial community.

When the alkaliphilic Fe(III)-reducing community was grown on solid phase Fe(III), 59 sequences were obtained from three different genera of bacteria. There are four different OTUs, three from the original population (5 Tissierella B sequences, 37 Tissierella C sequences and 2 Clostridium XI sequences) plus an OTU containing 15 Actinomycetales sequences (representative sequence KF798135).

### Analysis for of Soluble Electron-Shuttling Compounds

**Fig. 6:**
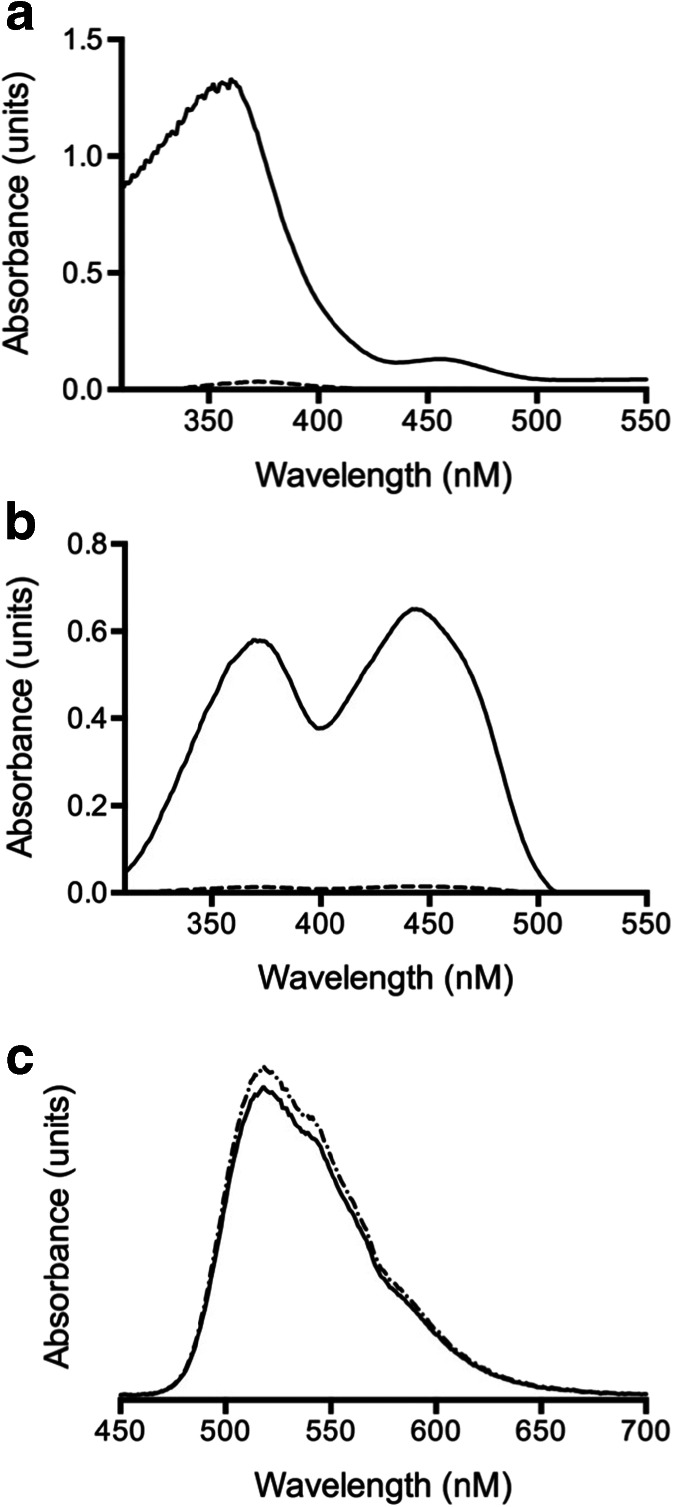
Spectral properties of spent aquifer sand medium after growth of the Fe(III)-reducing and Cr(VI)-medium communities. UV-visible spectra after **a** community growth and **b** isolation of extracellular compounds (*solid lines* are used for Fe(III)-reducing consortium, *dotted lines* for the Cr(VI)-medium communities). **c** Fluorescence spectrum of extracellular compounds isolated from Fe(III)-reducing community supernatant (chain-linked line) compared to that from commercial pure riboflavin (*solid line*). Upon excitation at 441 nm, the emission spectra were monitored between 450 and 700 nm

To investigate whether a soluble electron shuttling compound was involved in Fe(III) reduction, the spectral properties of the spent aquifer sand medium were studied. Two inoculums were used with aquifer sand medium: the original Fe(III)-reducing community and the *Clostridium XI* dominated, Cr(VI) medium community. Scanning the culture supernatants over a wavelength range of 300–550 nm revealed a large asymmetric double peak in the spent medium from the Fe(III)-reducing community, whereas there was only a very slight peak from the Cr(VI) medium community (Fig. 6a). Extraction of the extracellular compounds using an XAD-column showed a large double peak from the original Fe consortium, whereas there was a very minor peak associated with the Cr consortium (Fig. 6b). Upon excitation at 441 nm, the emission spectra from the extracellular compound from the Fe(III)-reducing community were found to be indistinguishable from those exhibited by commercially available riboflavin (Fig. 6c). Quantification of the flavin compounds revealed there to be approximately 0.11 μmol L^−1^ in the Fe(III)-reducing community supernatant and less than 0.01 μmol L^−1^ in the Cr(VI) medium community supernatant.

## Discussion

### Fe(III) Reduction Supports Growth of the Bacterial Community

The alkaliphilic Fe(III)-reducing community has evolved very little since the consortium was first cultivated by Whittleston et al. (2011), despite numerous further growth cycles in Fe(III)-citrate medium. Therefore, the population of this enrichment culture is considered stable when grown in this medium. It is dominated by a *Clostridium XI* and a *Tissierella* species (*Tissierella C*), which together form 80 % of the community (two other *Tissierella* and one *Alkaliphilus* species form the balance of the population; Fig. 5a). When cultured in Fe(III)-citrate medium, growth of the bacterial community is linked to Fe(III) reduction, and therefore, it is likely that one or more species in the community is able to use Fe(III) as the electron acceptor.

This bacterial community also grew without an exogenic electron acceptor (i.e. in the basal or Na-citrate media) presumably by utilising fermentable substrates in the yeast extract. A small number of clones from the base and Na-citrate media were sequenced for control purposes, and these showed that the main species were again *Tissierella C* and *Clostridium XI*, as in the original population. Comparison of the cell numbers when the bacterial community is grown in the three media (Fig. 2) clearly shows that the absence of Fe(III) from the medium results in less growth of this community (citrate did not have a significant impact on growth). However, whilst there is more growth in Fe(III)-containing medium, and this growth is coupled to its reduction to Fe(II), there is no direct evidence that the community uses Fe(III) as part of anaerobic respiration. Indeed, with a community of fermentative bacteria from the order *Clostridiales*, it is quite likely that Fe(III) reduction is being used by the cells to maintain internal redox balance during fermentative metabolism (Garnova et al. 2003; Kevbrin et al. 1998).

### Cr(VI) Puts a Selective Pressure the Bacterial Community

Attempts to grow the alkaliphilic Fe(III)-reducing community in Cr(VI) medium were only partially successful, with no growth in some replicates and attempts to culture-on the community failing completely after 10 growth cycles. Failure to get a community that grew reliably meant that growth could not be characterised in detail, but the time taken for complete removal of 200 μmol L^−1^ Cr(VI) from solution was very similar to the time taken for complete Cr(VI) removal from the FeCr200 medium (in both cases about ∼25 to 30 days). Growth in Cr(VI) medium puts a strong selective pressure on the original community, and after seven growth cycles, the population was dominated by the *Clostridium XI* species (Fig. 5c). This indicates that this is the most Cr(VI)-tolerant species in the original population; however, the fragile nature of the growth suggests that this bacterium can only just tolerate Cr(VI).

All the FeCr medium tests with initial Cr(VI) concentrations from 100 to 2000 μmol L^−1^ exhibited similar patterns in Cr(VI) removal, cell growth and Fe(III) reduction. Cr(VI) removal from solution started after about 20 days and was substantially complete before there was a significant change in the cell numbers. At the prevailing pH and Cr(VI) concentrations, the dominant aqueous Cr(VI) species is CrO_4_^2−^ (Richard and Bourg 1991), so sorption to cells was probably low because cell walls become increasingly negatively charged as pH increases (Ams et al. 2004). Also, Cr(VI) removal would be expected to start upon contact with the cells if the mechanism was sorption. It is therefore most likely that the bacterial community mediated the Cr(VI) removal from solution by a mechanism involving reductive transformation to Cr(III), which is predicted to precipitate as Cr(III) hydroxide at the prevailing pH value (Fendorf 1995) unless it is complexed by the citrate in the growth medium. Thus, Cr(VI) removal from FeCr medium appears to be associated with cell metabolism, but not with growth in cell numbers, probably because Cr(VI) inhibits cell growth.

Some anaerobic bacteria can reduce Cr(VI) as part of anaerobic respiration (Myers et al. 2000; Tebo and Obraztsova 1998), but frequently, it is a consequence of cellular uptake (Dhal et al. 2013). Chromate (CrO_4_^2−^) is isomorphous to sulphate (SO_4_^2−^) and easily crosses cell membrane of most bacteria to where it can be reduced to Cr(III) by cytoplasmic enzymes (Cervantes and Silver 1992), which is toxic as it forms stable bonds with protein and DNA in the cytoplasm (Costa 1997). Thus, the period when there was Cr(VI) reduction without apparent increase in cell numbers may be a period of fermentative cell growth balanced by cell death due to chromium toxicity. On the other hand, it has been shown that the community can grow fermentatively in the base medium and can reduce Fe(III) when it is present in the medium, probably as means of maintaining the fermentation redox balance. As Cr(VI) is readily reduced to Cr(III) by reoxidation of Fe(II) to Fe(III) (Richard and Bourg 1991), Cr(VI) reduction in FeCr medium may be the consequence of the onset of iron reduction (Fe(II) will only accumulate in the medium once all Cr(VI) has been reduced). The accumulation of Fe(II) in the FeCr medium immediately after exhaustion of Cr(VI) may seem to be indicative of the latter mechanism. However, this indirect mechanism involving Fe(II)/Fe(III) cycling cannot explain Cr(VI) reduction in Cr(VI) medium, where there was no source of Fe(III). Equally, the similar time taken to remove 200 μmol L^−1^ Cr(VI) from the Cr(VI) and FeCr200 media seems indicative of similar Cr(VI) reduction mechanisms in the two systems, suggesting either a cellular uptake mechanism or possibly direct Cr(VI) reduction as means of maintaining the fermentation redox balance (i.e. a similar mechanism to Fe(III) reduction). However, the data presented here cannot determine which is more likely mechanism of Cr(VI) reduction.

The final cell numbers after complete Fe(III) reduction were of similar order of magnitude in the FeCr and Fe(III)-citrate media. However, in the FeCr200 medium, the proportion of the population that were *Clostridium XI* and *Alkaliphilus* species increased, while the proportion that were *Tissierella* species was much reduced compared with the original population (the relative abundances of *Clostridium XI* and *Alkaliphilus* were similar in the two media). This change in community composition after a single growth cycle suggests that the *Tissierella* species have very low tolerance for Cr(VI).

The FeCr medium tests with very high initial Cr(VI) concentrations (4500 and 8500 μmol L^−1^) behaved differently from those containing lower concentrations. In the FeCr4500 medium, the removal of Cr(VI) from solution started after about 32 days and was only 60 % complete after 56 days, yet cell numbers started to increase after 39 days. In the FeCr8500 medium, there was very little change in the Cr(VI) concentration (it decreased by ∼10 % over the test), yet cell numbers started to increase after ∼60 days. The final microbial population in the FeCr8500 medium consisted of Brevibacterium and Actinomycetales bacteria (see Supplementary Information Fig. 1). These hardier species were presumably a very small component of the initial population that are not able to compete effectively with the main population when grown in Fe(III)-citrate medium but were able to grow fermentatively using yeast extract when there was extreme Cr(VI) stress, because these conditions were not conductive to growth of the other species.

### A Change in the State of Fe(III) Source Puts a Selective Pressure the Bacterial Community

When the alkaliphillic Fe(III)-reducing community was cultured in aquifer sand medium, Fe(III) reduction to Fe(II) became apparent after about 10 days, which is significantly slower than was observed with Fe(III)-citrate medium (where Fe(III) reduction was apparent after 4 days). The difference is probably associated with the availability of Fe(III) to the bacteria. In the aquifer sand, Fe(III) is present as Fe(III) oxyhydroxides in fine particles and grain coatings, which is probably less accessible to bacteria than an aqueous Fe(III)-citrate complex (in the absence of complexing ligands, such as citrate, Fe(III) is very sparingly soluble at pH values above pH8: Langmuir 1997). Growth in aquifer sand medium led to an increased abundance of *Tissierella C* species (from ∼1/3 to >2/3 of sequences). Growth of the community was also associated with the release of a riboflavin-like molecule into the aquifer sand medium. It has previously been shown that growth of the alkaliphilic Fe(III)-reducing community in Fe(III)-citrate medium results in the release of riboflavin contemporaneously with Fe(III) reduction and that the *Tissierella C* sp. can reduce Fe(III) remote from the cells when grown anaerobically on agar-Fe(III)-citrate medium plates (Fuller et al. 2014). Thus, it seems likely that the *Tissierella C* species can release riboflavin as an electron shuttling compound to facilitate extracellular electron transfer, and this may give it a competitive advantage when grown of aquifer sand medium.

Growth of the *Clostridium XI* dominated, Cr(VI)-medium community in aquifer sand medium did not result in any further change in the community composition, probably because community diversity was lost during repeated growth in Cr(VI) medium (populations C, E and F in Fig. 5). Also, while the increase in Fe(II) concentration started after a similar lag-time with the two populations, the amount of Fe(II) produced by the Cr(VI)-medium community after 50 days was about one third that produced by the alkaliphillic Fe(III)-reducing community (Fig. 4). Thus, the rate at which the Cr(VI)-medium community could reduce that Fe(III) is significantly slower than the *Tissierella C*-dominated community. No significant release of riboflavin was associated with growth of the Cr(VI)-medium community; thus, the absence of an electron shuttling compound may explain the slower rate of Fe(III) reduction. However, the Cr(VI)-medium community can clearly reduce Fe(III) when grown in aquifer sand medium, and thus, the *Clostridium XI* specie must possess a mechanism from transferring electrons external electron acceptors. Given the lower rate of Fe(III) reduction and the absence of an electron shuttling compound, it is speculated that Fe(III) reduction might involve direct contact between the cell and the iron bearing solid phase.

### Implications for Bioremediation Processes at COPR Impacted Sites

This paper clearly shows that bioremediation has great potential for the treatment of highly alkaline COPR impacted sites. Leachate from the historical COPR site from which the bacteria were cultivated has a Cr(VI) concentration of 1000 μmol L^−1^ within the waste and 200 μmol L^−1^ within the drainage ditch adjacent to the site (Stewart et al. 2010). This study shows that bacteria from the same site are able to grow and reduce chromate in solutions containing up to 2000 μmol L^−1^ Cr(VI) provided a source of Fe(III) is present. This exceeds the maximum leachate concentration reported for any COPR disposal site thus far. In most microcosms, the pH buffered downwards to around 8.5 before the exponential phase of growth indicating that the bacteria in this study are most comfortable at lower pH. This is interesting as other work has found that buffering the pH down to ∼9 results in more robust Fe(III) reduction in COPR-contaminated soils (Whittleston et al. 2013).

The pH of COPR leachate is high in comparison with the pH range used in this study, but it is quickly buffered by interactions with soil (Whittleston 2011). This study suggests that in bioremediation (i.e. by addition of labile organic matter to soils) should be successful in areas where this has occurred. Addition of acid or bicarbonate to increase pH buffering may further increase the likelihood of success. Indeed, where sufficient labile organic matter is naturally present, lowering the soil pH to ≤9 may be all that is required to initiate bioreduction (Whittleston et al. 2013).

Few bacterial species have been reported that can use Cr(VI) as an electron acceptor to support cell growth. Thus at a contaminated site, iron-reducing microorganisms are likely to outnumber Cr(VI) reducers, as iron-reducing microorganisms are numerous and widespread in nature (Lovley 2006). This is supported by the fact that only Clostridium XI in this study could grow weakly with Cr(VI) as an electron acceptor, and it grew far more robustly with Fe(III). Therefore, the accumulation of Fe(II) in soils and subsequent abiotic reduction of Cr(VI) by solid associated Fe(II) is probably the most important mechanism of Cr(VI) reduction at COPR sites.

However, it is also clear that several of the species present (e.g. the *Tissierella C* sp.) in the Fe(III)-reducing enrichment culture have a low tolerance for Cr(VI) despite being recovered from immediately beneath a COPR waste tip. This may indicate that micro-habitats are important on site, with species such as Tissierella restricted to local environments where Cr(VI) concentration is low. Indeed, the soluble electron shuttling compound produced by *Tissierella C* may be important to the creation of micro-habitats as it can facilitate Fe(III)-reduction remote from the cells, producing Fe(II) in the soils, which can indirectly remove Cr(VI) from the local environment around the cells.

## Conclusions

Growth and community structure of the consortium of alkaliphilic Fe(III)-reducing bacteria were significantly affected by changes in growth conditions. When Cr(VI) was added to the Fe(III)-citrate growth medium, the *Tissierella* sp. that were present in the original culture reduced in abundance and died out completely when the community was grown on Cr(VI) medium. Of the species present in the original community, the *Clostridium XI* sp. was most tolerant of higher Cr(VI) concentrations, but even this species only grew weakly in a Cr(VI) medium, dying out after only 10 growth cycles. Conversely, when solid phase Fe(III) oxyhydroxides were provided in the growth medium, the *Tissierella* C sp. increased in abundance relative to other bacteria in the initial community. This was possibly due to the *Tissierella* C sp. producing riboflavin as an extracellular electron shuttling compound allowing more efficient reduction of the solid Fe(III) phases present. The indigenous bacteria recovered from soils beneath COPR waste are shown to adopt several different strategies for survival in presence of an influx alkaline Cr(VI)-containing water; however, strategies involving Fe(III)-reduction produced more robust and reproducible growth. Stimulation of bacterial growth in the soils adjunct to COPR sites is therefore likely to create conditions where Fe(II) accumulation in those soils acts as an in situ barrier that reduces Cr(VI) mobility and toxicity.

## Electronic supplementary material

ESM 1 (DOCX 306 kb)
