# Supplementary material for: Population Changes in a Community of Alkaliphilic Iron-Reducing Bacteria Due to Changes in the Electron Acceptor: Implications for Bioremediation at Alkaline Cr(VI)-Contaminated Sites
Source: Water Air Soil Pollut. 2015 May 13;226(6):180. doi: 10.1007/s11270-015-2437-z (PMC4429135; doi:10.1007/s11270-015-2437-z)
Supplement: Supplementary file 1 — (DOCX 306 kb) [file 11270_2015_2437_MOESM1_ESM.docx]

**Supplementary Information for:**

Population Changes in a Community of Alkaliphilic Iron Reducing Bacteria Due to Changes in the Electron Acceptor: Implications for Bioremediation at Alkaline Cr(VI) Contaminated Sites

*Samuel J. Fuller^a^, Ian T. Burke^b*^, Duncan G. G. McMillan^c^, Weixuan Ding^d^, Douglas I. Stewart^a*^*

^a^ School of Civil Engineering, University of Leeds, Leeds, LS2 9JT, UK

^b^ School of Earth and Environment, University of Leeds, Leeds LS2 9JT, UK

^c^ University Hospital Jena, Friedrich-Schiller University, Jena, 07743, Germany

^d^ School of Process, Environmental and Materials Engineering, University of Leeds, Leeds, LS2 9JT, UK

* Corresponding Authors: [d.i.stewart@leeds.ac.uk](mailto:d.i.stewart@leeds.ac.uk) and [i.t.burke@leeds.ac.uk](mailto:i.t.burke@leeds.ac.uk).

This section consists of 3 pages containing 2 figures.

**Further data on the growth of alkaliphilic iron reducing community in FeCr media**

Figure S1. Growth of alkaliphilic Fe(III) reducing community in FeCr medium with initial Cr(VI) concentrations of 100, 150, 500 and 4500 µmol.L^-1^. (A) Cr(VI) concentration with time (B) Total Fe(II) concentration with time (C) cell numbers with time (D) pH with time (data shown is complementary to that shown in Figure 3).

Figure S2: Percentage of bacteria sequences recovered from FeCr8500 medium assigned to each OTU.
